# Disparities in multidimensional psychosocial stressors by sexual identity among cancer survivors from the All of Us Research Program

**DOI:** 10.1007/s10552-026-02157-w

**Published:** 2026-04-03

**Authors:** Angel Arizpe, Nikta Saeedi, Carol Y. Ochoa-Dominguez, Theresa A. Hastert, Alberto Carvajal, Sue E. Kim, Albert J. Farias

**Affiliations:** 1 Keck School of Medicine of the University of Southern California, Los Angeles, CA USA; 2 University of California, San Diego, San Diego, CA USA; 3 Department of Oncology, Wayne State University School of Medicine, Detroit, MI USA; 4 Department of Population and Health Sciences, Keck School of Medicine of the University of Southern California, 1845 N. Soto St., Suite 318B, Los Angeles, CA 90032 USA

**Keywords:** Sexual minorities, Cancer survivorship, Stress, Social cohesion, Medical discrimination

## Abstract

**Objectives:**

To examine disparities in psychosocial stressors by sexual identity among cancer survivors and whether these differ by state governmental leaning.

**Methods:**

Cross-sectional data (2018–2022) from 14,806 cancer survivors in the All of Us repository were analyzed. Sexual identity (heterosexual vs. sexual minority) was examined in relation to discrimination in medical settings (DMS), perceived stress (PS), and neighborhood social cohesion (NSC) using multivariable logistic regression models adjusted for sociodemographic and clinical covariates, stratified by state governmental leaning.

**Results:**

6.3% of survivors identified as sexual minority (SM). Compared with heterosexual survivors, SM individuals had higher odds of medium/high perceived stress (aOR = 1.46, 95% CI 1.26–1.72) and low neighborhood social cohesion (aOR = 1.47, 95% CI 1.27–1.71). In Republican-leaning states, they had over twice the odds of medical discrimination (aOR = 2.31; 95% CI = 1.50–3.71), with no association in Democratic-leaning states.

**Conclusions:**

Sexual minority cancer survivors experience greater psychosocial stressors regardless of their state’s governmental leaning, which may impact their cancer survivorship. Those in Republican-leaning states are more likely to have DMS experiences, potentially affecting their access to care and overall health. Future research should explore the long-term effects of policy environments and their influence on discrimination and psychosocial stress among SM cancer survivors.

**Supplementary Information:**

The online version contains supplementary material available at 10.1007/s10552-026-02157-w.

## Introduction

Sexual minority (SM) individuals, who comprise an estimated 9.3% of the United States population [1], experience significant health disparities compared to their heterosexual counterparts, largely due to systemic barriers in healthcare access, stigma, and discrimination [2]. The minority stress model is a framework that suggests that chronic exposure to prejudice, social rejection, and internalized stigma contributes to worse health outcomes for SM populations [2]. Studies have shown that SM patients often delay or avoid medical care because of prior negative experiences or fears of mistreatment from providers [3]. In a national survey from 2011, nearly 8% of SM adults reported being denied care, while many others described hostility or dismissal of their health concerns [4].

Chronic stress from cumulative systemic discrimination has been linked to both physiological and psychological harm. For instance, discrimination-related stress has been found to be associated with elevated inflammatory markers and cardiovascular risk [5]. In addition, prolonged stress may lead to harmful health behaviors, such as increased substance use, poor diet, and avoidance of medical care, all of which can compound negative health outcomes [6]. These mechanisms are particularly important to consider in the context of serious or chronic illness, like cancer, where stress and behavior may directly influence disease progression and treatment outcomes.

As such, it makes sense that sexual minority cancer survivors face unique challenges that are shaped not only by the general burdens of cancer survivorship but also by the added effects of stigma, discrimination, and minority stress. There are over 18.1 million cancer survivors in the United States, many of whom face the physical, emotional, and financial burdens of ongoing treatment and monitoring [7]. The frequency of medical appointments and the long-term treatments required for survivorship may exacerbate chronic stressors like societal marginalization, particularly when SM survivors are reluctant to engage with healthcare due to prior experiences of discrimination [8]. Social isolation may further compound these challenges. Older SM survivors—particularly gay and bisexual men—are more likely to live alone and report social isolation [9], which has been associated with later-stage diagnoses and poorer cancer survival outcomes [10]. Additionally, survivorship experiences may differ by sex. Prior research has documented sex differences in cancer survivorship experiences, with female survivors reporting higher levels of psychological distress and fatigue, as well as greater symptom burden and worse physical and emotional functioning, compared to male survivors [11, 12].

Beyond individual experiences, the broader social and governmental contexts in which SM survivors reside may influence their access to care and psychosocial well-being, as state-level policies and societal attitudes can create environments that either mitigate or exacerbate challenges for SM populations. While it is well established that stigma, discrimination, and psychosocial stressors negatively impact healthcare access and outcomes for SM individuals, there is limited understanding of these associations in the context of cancer survivorship. Furthermore, to our knowledge, no studies have assessed how these disparities differ based on broader political environments. Thus, this study aimed to estimate associations between sexual minority identity and psychosocial stressors among cancer survivors from the All of Us program and explore whether these associations differ by state socio-political climates as measured by state governmental leaning.

## Method

### Data collection and sample

We assessed cross-sectional survey data from May 2018 to July 2022 from the All of Us (AoU) program. Participants enrolled in the AoU program signed a consent form for data collection following the Declaration of Helsinki protocol. Data for this study were de-identified and made available to AoU-approved researchers. The All of Us program was approved by the National Institutes of Health (NIH) Institutional Review Board (IRB). This study was deemed exempt ‘non-human subjects’ research, with no ethical approval or participant consent required, as confirmed by the USC Institutional Review Board.

Our cohort included participants who reported that they were ever told by their healthcare provider that they had/have cancer. We excluded participants with missing data on self-reported discrimination in the medical setting, social neighborhood cohesion, and perceived stress scales (*n* = 15,568); those with multiple cancers (*n* = 6174); and those with missing sexual orientation status (*n* = 598), which leads to a final analytical sample of *n* = 14,806.

### Measures

#### Exposures

SM identity: Using a single question that asked participants to describe the best representation of how they think of themselves [13], we created a binary indicator of SM identity where those who self-identified as heterosexual were coded as heterosexual and those who self-identified as bisexual, gay, lesbian, queer, asexual, two-spirit, polysexual, omnisexual, sapiosexual or pansexual were coded as SM identity individuals.

#### Outcomes

Discrimination in Medical Settings (DMS) is an adapted 7-item scale [14] from the Everyday Discrimination Scale (EDS) [15], that assesses the participants’ prior treatment experiences while getting healthcare services. This measure captures general experiences of discrimination in healthcare settings and is not limited to cancer-related care or a specific time frame. Participants were asked, “How often do any of these (perceived discriminatory events) happen to you when you go to a doctor’s office or other health care provider? Example items include “You feel like a doctor or nurse is not listening to what you were saying”, “A doctor or nurse acts as if he or she thinks you are not smart.”, “A doctor or nurse acts as if he or she is afraid of you”. Responses were measured on a 5-point Likert scale, ranging from never (0) to always (4). We created a dichotomized indicator of never vs. any DMS, where if participants selected never having experienced DMS in all seven items, they were then coded as never and were set as the reference. Similar methods of dichotomizing each question have been assessed previously using this measure [16].

Neighborhood Social Cohesion (NSC) was measured using a 4-item scale that quantifies an individual’s perception of their neighborhood, including trust and social relationships with others in their community [17], which are important for accessing resources and buffering stress. This measure reflects participants’ current perceptions of their neighborhood environment and is not tied to a specific time frame. Example items include “People in my neighborhood generally get along with each other” and “People in my neighborhood share the same values”. Responses were measured on a 5-point Likert scale, which ranged from “strongly agree (1)” to “strongly disagree (5)”. We created a summed score from those questions that ranged from 4 to 20 and then dichotomized the measure at the median, consistent with prior studies [18]. Scores below the median were classified as low NSC, whereas scores at or above the median score were classified as high NSC.

Perceived Stress (PS) was measured using the 10-item Perceived Stress Scale, which asked participants about their feelings and thoughts in the past month. Example items include “In the last month, how often have you been upset because of something that happened unexpectedly?”, “In the last month, how often have you felt difficulties were piling up so high that you could not overcome them?”. Responses were measured on a 5-point Likert scale, from never to very often. We created a summed score that ranged from 0 to 40 and dichotomized it using recommended cutoffs. Scores of 0–13 were categorized as low PS and scores ≥ 14 as moderate/high PS [19].

Demographics and covariates: Demographic self-reported characteristics included in our study were continuous age (measured at the time of survey completion), biological sex (male vs. female), race/ethnicity (non-Hispanic White vs. categories: non-Hispanic Asian, non-Hispanic Black, Hispanic, and, Other [includes: more than one race, another race, and none of these]), marital status (married [includes: living with a partner] vs single [includes: single, divorced, widowed, and separated]), active cancer treatment (yes vs. no), and nativity status (US-born vs foreign-born). Socioeconomic status (SES) was assessed using a SES barrier index made of five SES factors: education (≤ high school), income (≤ $35,000 which are those in the lowest quantile), insurance (none), housing (rent/other), and employment status (not employed). The summed score ranged from 0 to 5 and was truncated to 3 + due to sparsity. Higher scores in this SES index indicate higher SES barriers [20].

#### Moderator

Using data from the National Governor’s Association (2023) [21], we created a binary indicator of state governmental leaning. State governmental leaning was defined by the political affiliation of the sitting governor as Democrat or Republican during the time of survey completion. We used self-reported state residential information and assigned individuals to their respective state’s governmental leaning. For example, if they resided in a state with a Democratic governor, they were categorized as living in a Democratic-leaning state.

#### Statistical methods

Descriptive statistics used Chi-square or Mann–Whitney U tests to determine the association of all the variables with the exposure (SM) and outcomes (DMS, NSC, PS) separately. Multivariable logistic regression models assessed whether SM identity was associated with our outcomes of interest. Interaction terms were included in the models to explore whether the SM identity and outcomes differed by state governmental leaning (SM*state governmental leaning) and sex (SM*sex). Covariates included in multivariable models were age, sex, nativity, SES barriers index, 2023 state governmental leaning, PS, DMS, or NSC, and active treatment status. All statistical analyses were performed using R Jupyter Notebooks accessed via the “All of Us” workbench and using a significance level at alpha < 0.05. Adjusted odds ratios (aORs) with 95% confidence intervals (CI) and p-values were reported.

## Results

Our final analytical sample consisted of 14,806 cancer survivors with a median age of 69 (Interquartile range [IQR(Q1, Q3)] = 59.9, 74.6) years. Most participants identified as non-Hispanic White as their race/ethnicity (88%) and reported their biological sex as female (61%). The majority of cancer survivors reported being married (68%), US-born (93%), and having experienced any DMS (72%). Approximately half reported low PS (52%) and low NSC (49.5%) (Table 1). Cancer survivors who self-identified as SM had a higher prevalence of ever experiencing DMS (80% vs 72%), medium/high PS (62% vs 47%), and low NSC (64% vs 48%) compared with heterosexual survivors (Table 2).

**Table 1:** Demographic characteristics of All of Us cancer survivors by sexual orientation status

	Heterosexual	Sexual minority	*p*	Total
	(*N* = 13,867) *n* (%)	(*N* = 939) *n* (%)		(*N* = 14,806) *n *(%)
Race/ethnicity			0.02	
Non-hispanic white	12,244 (88.3)	826 (88.0)		13,070 (88.3)
Hispanic	670 (4.8)	58 (6.2)		728 (4.9)
Non-hispanic black	505 (3.6)	30 (3.2)		535 (3.6)
Non-hispanic Asian	> 100 (> 0.5)	≤ 20 (< 1.0)		145 (1.0)
Other	> 80 (> 0.5)	≤ 20 (< 1.5)		104 (0.7)
Missing	> 180 (> 1.0)	≤ 20 (< 1.0)		224 (1.5)
Sex			< 0.001	
Male	> 5000 (> 35.0)	< 500 (< 55.0)		5675 (38.3)
Female	8607 (62.1)	437 (46.5)		9044 (61.1)
Missing	> 80 (> 0.5)	≤ 20 (< 1.0)		87 (0.6)
Age			< 0.001	
Mean (SD)	66.2 (11.7)	60.7 (13.9)		65.8 (11.9)
Median [Q1, Q3]	68.4 [59.7, 74.2]	63.6 [52.5, 70.6]		67.9 [59.6, 74.4]
Income status			< 0.001	
Q1 (lowest income)	2997 (21.6)	321 (34.2)		3318 (22.4)
Q2-Q5	10,870 (78.4)	618 (65.8)		11,488 (77.6)
Marital status			< 0.001	
Married	> 9500 (> 65.0)	< 600 (< 55.0)		10,079 (68.1)
Single	4205 (30.3)	415 (44.2)		4620 (31.2)
Missing	> 80 (> 0.5)	≤ 20 (1.4)		107 (0.7)
Education status			0.65	
Some college +	> 12,000 > (90.0)	< 900 (< 95.0)		13,558 (91.6)
≤ HS	1077 (7.8)	67 (7.1)		1144 (7.7)
Missing	> 80 (> 0.5)	≤ 20 (0.4)		104 (0.7)
Insurance status			0.36	
Insured	13,648 (98.4)	918 (97.8)		14,566 (98.4)
Uninsured	> 100 (> 0.5)	≤ 20 (< 2.0)		141 (1.0)
Missing	> 80 (> 0.5)	≤ 20 (< 1.0)		99 (0.7)
Nativity			0.63	
US-born	> 12,500 (> 90.0)	< 900 (< 95.0)		13,831 (93.4)
Foreign-born	864 (6.2%)	50 (5.3%)		914 (6.2%)
Missing	> 45 (> 0.1%)	≤ 20 (< 1.0%)		61 (0.4%)
Housing status			< 0.001	
Own	> 11,000 (> 80.0)	< 600 (< 65.0)		11,821 (79.8)
Rent/other arrangement	2493 (18.0)	346 (36.8)		2839 (19.2)
Missing	> 100 (> 0.5)	≤ 20 (< 1.0)		146 (1.0)
Employment status			< 0.001	
Employed	> 12,000 (> 90.0)	< 800 (< 85.0)		13,434 (90.7)
Not employed	1127 (8.1)	136 (14.5)		1263 (8.5)
Missing	> 80 (> 0.5)	≤ 20 (< 1.0)		109 (0.7)
Governor political affiliation			0.15	
Democrat	> 11,000 (> 80.0)	< 800 (< 80.0)		11,858 (80.1)
Republican	2709 (19.5)	205 (21.8)		2914 (19.7)
Missing	> 20 (> 0.1)	≤ 20 (< 0.1)		34 (0.2)
Active treatment			0.02	
No	> 10,000 (> 70.0)	< 800 (< 80.0)		10.911 (73.7)
Yes	3643 (26.3)	206 (21.9)		3849 (26.0)
Missing	> 20 (> 0.1)	≤ 20 (< 1.0)		46 (0.3)
Socioeconomic barrier			< 0.001	
0	9497 (68.5)	487 (51.9)		9984 (67.4)
1	2795 (20.2)	244 (26.0)		3039 (20.5)
2	973 (7.0)	111 (11.8)		1084 (7.3)
3 +	602 (4.3)	97 (10.3)		699 (4.7)
Discrimination in medical settings			< 0.001	
Never	3941 (28.4)	190 (20.2)		4131 (27.9)
Any	9926 (71.6)	749 (79.8)		10,675 (72.1)
Perceived stress			< 0.001	
Low	7385 (53.3)	354 (37.7)		7739 (52.3)
Medium/high	6482 (46.7)	585 (62.3)		7067 (47.7)
Social neighborhood cohesion			< 0.001	
Better	7149 (51.6)	334 (35.6)		7483 (50.5)
Low	6718 (48.4)	605 (64.4)		7323 (49.5)

Married includes living with a partner, Single includes Divorced, Widowed, and Separated

Per "All of Us" data use agreement policy, groups < 20 participants are shown as ≤ 20 (%) with a corresponding > (%) category to prevent deriving counts < 20 from other values

Not all percentages equal to 100

Chi-square or Fisher tests were performed to obtain p-values (*p*)

Income: Lowest Quintile: includes individuals with income of ≤ $35 K

**Table 2:** Demographic characteristics of All of Us cancer survivors by types of stress indicators

	Discrimination in medical settings	*p*	Perceived stress	*p*	Neighborhood social cohesion	*p*
	Any		Medium/High		Low	
	(*N* = 10,675) 72.1% *n* (%)		(*N* = 7,067) 47.7% *n* (%)		(*N* = 7,323) 49.5% *n *(%)	
Race/ethnicity		0.35		< 0.001		< 0.001
Non-hispanic white	> 9000 (> 70.0)		6085 (46.6)		6254 (47.9)	
Hispanic	529 (72.2)		454 (62.4)		456 (62.6)	
Non-hispanic black	392 (73.3)		285 (53.7)		364 (68.0)	
Non-hispanic Asian	107 (73.8)		80 (55.2)		74 (51.0)	
Other	< 100 (< 85.0)		66 (63.5)		64 (61.5)	
Missing	160 (71.4)		97 (43.3)		111 (49.6)	
Sex		< 0.001		< 0.001		0.16
Male	3764 (66.3)		2167 (38.2)		2740 (48.3)	
Female	6848 (75.7)		4853 (53.7)		4541 (50.2)	
Missing	63 (72.6)		47 (54.0)		42 (48.3)	
Sexual minority status		< 0.001		< 0.001		< 0.001
Heterosexual	9926 (71.6)		6482 (46.7)		6718 (48.4)	
Sexual minority	749 (79.8)		585 (62.3)		605 (64.4)	
Age		< 0.001		< 0.001		< 0.001
Mean (SD)	65.2 (12.1)		62.5 (12.9)		64.6 (12.5)	
Median [Q1, Q3]	67.5 [58.6, 73.6]		64.6 [54.6, 72.0]		66.8 [57.6, 73.6]	
Income		< 0.001		< 0.001		< 0.001
Q1 (lowest income)	2560 (77.2)		1998 (60.2)		2080 (62.7)	
Q2-Q5	8115 (70.6)		5069 (44.1)		5243 (45.6)	
Marital status		< 0.001		< 0.001		< 0.001
Married	7,112 (70.6)		4,518 (44.8)		4,531 (45.0)	
Single	3,483 (75.4)		2,496 (54.0)		2,733 (59.2)	
Missing	80 (74.8)		53 (49.5)		59 (55.1)	
Education		0.04		< 0.001		< 0.001
Some college +	9,819 (72.4)		6,361 (46.9)		6,589 (48.6)	
≤ HS	786 (68.7)		652 (57.0)		683 (59.7)	
Missing	70 (67.3)		54 (51.9)		51 (49.0)	
Insurance status		0.35		< 0.001		< 0.001
Insured	10,490 (72.0)		6,913 (47.5)		7,178 (49.3)	
Uninsured	107 (76.0)		95 (67.4)		86 (61.0)	
Missing	78 (78.8)		59 (59.6)		59 (59.6)	
Nativity status		0.42		0.86		< 0.001
USA-born	9,979 (72.1)		6,589 (47.6)		6,777 (49.0)	
Non-US-born	647 (70.8)		449 (49.1)		518 (56.7)	
Missing	< 50 (80.3)		29 (47.5)		28 (45.9)	
Housing status		< 0.001		< 0.001		< 0.001
Own	8,393 (71.0)		5,168 (43.7)		5,284 (44.7)	
Rent/other arrangement	2,171 (76.5)		1,823 (64.2)		1,957 (68.9)	
Missing	111 (76.0)		76 (52.1)		82 (56.2)	
Employment status		< 0.001		< 0.001		< 0.001
Employed	9,576 (71.3)		6,085 (45.3)		6,427 (47.8)	
Not employed	1,025 (81.2)		921 (72.9)		826 (65.4)	
Missing	74 (67.9)		61 (56.0)		70 (64.2)	
Governor political affiliation		0.18		0.36		< 0.001
Democrat	> 8,400 (> 70.0)		> 5,500 (> 45.0)		> 5,500 (> 75.0)	
Republican	2,149 (73.7)		1,434 (49.2)		1,507 (73.2)	
Missing	≤ 20 (< 75.0)		≥ 20 (< 50.0)		≥ 20 (> 60.0)	
Active treatment		0.13		0.21		0.89
No	> 7,500 (> 70.0)		5,126 (47.0)		5,376 (49.3)	
Yes	2,719 (70.6)		1,920 (42.9)		1,925 (50.0)	
Missing	< 25 (< 70.0)		21 (45.7)		22 (47.8)	
Socioeconomic barrier		< 0.001		< 0.001		< 0.001
0	7,034 (70.5)		4,167 (41.7)		4,315 (43.2)	
1	2,252 (74.1)		1,626 (53.5)		1,741 (57.3)	
2	835 (77.0)		731 (67.4)		719 (66.3)	
3 +	554 (79.3)		543 (77.7)		548 (78.4)	
Discrimination in Medical settings				< 0.001		< 0.001
Never	-		1,213 (29.4)		1,629 (39.4)	
Any	-		5,854 (54.8)		5,694 (54.4)	
Perceived stress		< 0.001				< 0.001
Low	4,821 (62.3)		-		3,202 (41.4)	
Medium/high	5,854 (82.8)		-		4,121 (58.3)	
Social Neighborhood cohesion		< 0.001		< 0.001		
Better	2,901 (79.8)		2,275 (62.6)		-	
Low	7,774 (69.6)		4,792 (42.9)		-	

*SES* Socioeconomic, Married includes living with a partner, Single includes Divorced, Widowed, and Separated

Per "All of Us" data use agreement policy, groups < 20 participants are shown as ≤ 20 (%) with a corresponding > (%) category to prevent deriving counts < 20 from other values

No all percentages equal to 100

Chi-square or Fisher tests were performed to obtain p-values (*p*)

Income: Lowest Quintile: includes individuals with income of ≤ $35 K

*N* = 14,806

### SM and stressors

Adjusting for the state governmental leaning and other covariates, results from the multivariable models showed that compared to heterosexual cancer survivors, those who were SM individuals had a 34% (aOR = 1.36, 95% CI 1.15–1.63), 46% (aOR = 1.46, 95% CI 1.26–1.72) and 47% (aOR = 1.47, 95% CI 1.27–1.71) greater likelihood of reporting any DMS, high/moderate PS, and low NSC, respectively (Table 3).

**Table 3:** Multivariable association of sexual minority identity and stressors among All of Us cancer survivors

	Any Discrimination in medical settings	Medium/high perceived stress	Low Neighborhood social cohesion
	aOR (95% CI)	aOR (95% CI)	aOR (95% CI)
Variables			
Sexual orientation			
Heterosexual	Ref	Ref	Ref
Sexual minority	**1.34 (1.13–1.60)**	**1.46 (1.25–1.70)**	**1.47 (1.27–1.71)**
Race/ethnicity			
Non-hispanic white	Ref	Ref	Ref
Hispanic	**0.82 (0.68–0.99)**	1.17 (0.97–1.40)	**1.26 (1.06–1.50)**
Non-hispanic black	0.89 (0.72–1.10)	0.74 (0.61–0.91)	**1.63 (1.33–1.99)**
Non-hispanic Asian	1.04 (0.70–1.57)	1.13 (0.78–1.64)	0.92 (0.65–1.34)
Other	1.63 (0.98–2.87)	1.48 (0.96–2.32)	1.36 (0.90–2.08)
Sex			
Male	Ref	Ref	Ref
Female	**1.37 (1.27–1.49)**	**1.44 (1.33–1.55)**	0.84 (0.78–0.91)
Age	1.00 (0.99–1.00)	**0.96 (0.96–0.97)**	**0.99 (0.99–1.00)**
Governor political affiliation			
Democrat	Ref	Ref	Ref
Republican	**1.11 (1.01–1.22)**	0.99 (0.91–1.09)	1.04 (0.95–1.13)
Nativity status			
US-Born	Ref	Ref	Ref
Non-US-Born	0.94 (0.80–1.12)	**0.83 (0.71–0.98)**	**1.22 (1.13–1.44)**
Active treatment			
No	Ref	Ref	Ref
Yes	**0.89 (0.82–0.97)**	**1.13 (1.04–1.23)**	0.99 (0.92–1.08)
Socioeconomic barrier			
0	Ref	Ref	Ref
1	0.97 (0.88–1.07)	**1.36 (1.24–1.49)**	**1.51 (1.38–1.65)**
2	0.94 (0.80–1.11)	**2.13 (1.83–2.48)**	**1.87 (1.62–2.16)**
3 +	0.90 (0.79–1.12)	**2.87 (2.33–3.54)**	**2.99 (2.45–3.66)**

Adjusted for governor political affiliation, Socioeconomic barriers, age, marital status, nativity, active treatment, biological sex, social cohesion, perceived stress

*aOR* adjusted odds ratios, *CI* confidence interval

Bolded represents statistical significance

*N* = 14,276

In models stratified by the state’s governmental leaning, we observed no difference in associations between SM identity and perceived stress (p_interaction_ = 0.96) or neighborhood social cohesion (p_interaction_ = 0.97). Associations between SM identity and experiences of medical discrimination were stronger for cancer survivors living in Republican-leaning states (aOR: 2.31, 95% CI 1.50–3.71) compared with survivors living in Democratic-leaning states (aOR: 1.20, 95% CI 0.99–1.45; p_interaction_ = 0.001) (Table 4).

**Table 4:** Multivariable association between sexual minority status and discrimination in medical settings by state political party and sex

	Democratic Gov (*n* = 11,963) aOR (95% CI)	Republican Gov (*n* = 2313) aOR (95% CI)	Male (*n* = 5491) aOR (95% CI)	Female (*n* = 8785) aOR (95% CI)	
Heterosexual	Ref	Ref	Ref	Ref	
Sexual minority	1.20(0.99–1.45)	**2.31(1.50–3.71)**	1.10(0.88–1.38)	**1.90(1.42–2.58)**	

Adjusted for race, Socioeconomic barriers, age, marital status, nativity, active treatment, social cohesion, perceived stress

*aOR* adjusted odds ratios, *CI* confidence interval

Bolded represents statistical significance

We also tested for interaction between SM identity and biological sex. A significant interaction was observed for discrimination in medical settings (p_interaction_ = 0.01). In sex-stratified analyses, sexual minority women had significantly higher odds of reporting DMS compared to heterosexual women (aOR 1.90, 95% CI 1.42–2.58), whereas sexual minority men did not have significantly higher odds compared to heterosexual men (aOR: 1.10, 95% CI 0.88–1.38). No significant interaction was observed for sexual minority identity and sex for PS (p_interaction_ = 0.06) or NSC (p_interaction_ = 0.30).

## Discussion

Using data from the All of Us Research Program, this study found that SM cancer survivors had significantly higher odds of experiencing DMS, moderate/high PS, and low NSC compared to their heterosexual counterparts. While state governmental leaning did not significantly moderate the associations of SM identity status with PS or NSC, we observed a significant interaction among those residing in a Republican-leaning state and SM identity status and DMS. Specifically, SM identity was associated with more than twice the odds of experiencing DMS compared to heterosexual cancer survivors among those residing in Republican-leaning states, while SM identity was associated with a non-significant 20% higher odds of DMS among survivors living in Democratic-leaning states. These findings highlight the disproportionate burden of psychosocial stressors faced by SM cancer survivors and suggest that broader sociopolitical factors may play a role in shaping these experiences.

### Discrimination in the medical setting

Our findings indicated that SM cancer survivors had higher odds of reporting DMS. Discrimination and stigma contribute to social isolation, and they also discourage help-seeking behaviors, which can lead to delays in medical care and worse health outcomes. Studies have shown that SM individuals who perceive lower levels of community support are less likely to engage in routine healthcare and cancer screenings, thereby increasing their risk of late-stage diagnoses and poorer prognoses [3]. Prior research has established that experiences of DMS can lead to medical mistrust and avoidance of healthcare, ultimately resulting in delayed diagnoses and poorer prognoses for sexual minority individuals [22]. Additionally, stress from discrimination has been linked to chronic physiological dysregulation, resulting in higher levels of inflammation and cardiovascular risk, further exacerbating health disparities [5]. With respect to cancer survivorship, lower levels of social cohesion and support may further exacerbate these disparities, as strong social networks have been shown to improve adherence to treatment and overall health outcomes. [23]

Descriptive findings indicated that women overall reported higher levels of discrimination in medical settings compared to men (Table 2). In sex-stratified analyses we found that the association between sexual minority status and discrimination in medical settings was driven primarily by sexual minority women. Sexual minority women had significantly higher odds of reporting discrimination compared to heterosexual women, whereas no significant difference was observed among men. This finding aligns with literature documenting gender differences in healthcare experiences and psychosocial vulnerability among cancer survivors [11, 24]. It is possible that this intersection between gender and sexual minority status may compound exposure to stigma within medical settings, warranting further investigation.

### Social cohesion among sexual minority cancer survivors

Our findings suggest that SM cancer survivors have significantly higher odds of reporting low NSC compared to their heterosexual cancer survivor counterparts. This finding aligns with prior research suggesting that SM individuals experience differences in their perceptions of social cohesion and reinforces the importance of social networks for this vulnerable group. One study found that lesbian, gay, and bisexual adults were less likely to feel that their neighborhood was close-knit, to trust their neighbors, or to believe neighbors helped each other out, even after adjusting for socio-demographic characteristics, living arrangements, health status, region, and neighborhood tenure [25]. Although we found lower levels of NSC within our study cohort, neighborhood social cohesion is also associated with fewer psychological symptoms and serves as a protective factor against negative health outcomes, including myocardial infarction and stroke [26, 27]. One study notes that even perceived social support may also be more critical than actual support available or received in terms of quality of life outcomes, such as reduced depressive symptoms, lower levels of distress, and improved mental health [28, 29].

### State governmental leaning

State governmental leaning significantly moderated the association between SM identity status and discrimination in medical settings (DMS) for SM cancer survivors, it did not moderate the associations between SM identity status and PS or NSC. This aligns with research that specific policy environments can directly influence how SM individuals experience healthcare. State policies such as employment nondiscrimination protections can protect against minority stress, while exclusionary measures like antigay marriage amendments exacerbate minority stress by limiting access to resources and increasing exposure to stigma [30]. Similarly, the presence or absence of nondiscrimination and religious exemption laws is closely tied to mental health outcomes among SM adults, wherein states without these protections or with broad exemptions are significantly associated with higher levels of anxiety and depression [31]. In our study, the link between state governmental leaning and discrimination in healthcare likely reflects a similar mechanism, as state leadership can play a key role in determining whether protective or harmful policies are enacted. This would make medical discrimination more directly tied to a state’s political leadership.

The lack of association by gubernatorial party affiliation between SM minority identity and PS or NSC in our study, however, may reflect a more multifactorial nature of PS and NSC. PS and NSC are also likely shaped by personal, community, and cultural factors that extend beyond state-level leadership, as these outcomes develop over longer periods and may be more of a response to nationwide sociocultural trends or movements. While policies matter, the ways through which they influence outcomes, such as their visibility, enforcement, and cumulative exposure over time, are complex and not always experienced uniformly [31]. Ultimately, our findings highlight that while political leadership may be a key driver of DMS, psychosocial outcomes like PS and NSC are broader and may require more nuanced, qualitative models to fully understand how they are associated with a state’s political environment.

### Strengths and limitations

To our knowledge, this is the largest study on SM identity and psychosocial stressors among cancer survivors in the U.S. Harnessing data from the All of Us research program, we assessed the independent associations of SM identity status and different types of stressors (DMS, PS, NSC) in a large sample of U.S. adult cancer survivors. A key strength of this study is the use of a large dataset that enhances the sample size of sexual minorities and thus the generalizability of our findings, as we have a range of survivors from geographically diverse areas in the US. By incorporating state-level governmental context, to our knowledge, this is the first study to examine how state-level political context may shape the experiences of SM cancer survivors. Furthermore, the inclusion of multiple psychosocial measures allows for a more nuanced analysis of distinct aspects of social determinants of health and provides a comprehensive understanding of their impact on this population.

However, this study is not without limitations. We excluded a slightly higher proportion of racial/ethnic minorities and those with two or three or more SES barriers because they did not have complete information on psychosocial stressors, which could have introduced potential for selection bias towards the null. Thus, as the AoU programs continue to enroll participants and those enrolled complete surveys, future studies should reassess this relationship with more complete and representative data. An additional limitation is that data were only available on sexual identity of self and not as a desired attribute in a partner. Thus, individuals could be male but prefer pansexual or sapiosexual partners, for example and not fit into either gay, lesbian or bisexual. We were also unable to disaggregate sexual minority (e.g., gay vs heterosexual, lesbian vs heterosexual) cancer survivors due to a small sample size (93.7% heterosexual, 2.3% bisexual, 2.7% gay, 1.2% lesbian, and 0.2% other) in full adjusted models. To address this limitation, we explored these relationships in unadjusted models (Supplementary Table 1) and models adjusted for stressors (Supplementary Table 2), highlighting that bisexual cancer survivors, compared to heterosexuals, experienced all three stressors, whereas the other groups experienced only two, indicating an area for future investigation. We were also unable to assess gender identity in this study, as in our cohort, only 33 cancer survivors identified as transgender men or women in the sexual orientation question and were excluded from our study, underscoring the need for research in this population.

An additional limitation is that the DMS measure captures general experiences of discrimination in healthcare and is not specific to cancer-related care. As such, we are unable to determine whether reported discrimination occurred during cancer treatment or in other healthcare contexts. However, discrimination in any healthcare setting may limit engagement in preventive services and ongoing care, potentially affecting cancer survivors at multiple critical timepoints across the cancer care continuum (screening/prevention, diagnosis, treatment, and survivorship care).

We also recognize that state governmental leaning was assigned based on participants’ current state of residence at the time of survey completion. Because the discrimination in medical settings measure reflects overall cumulative healthcare experiences, some reported discrimination may have occurred in states other than the participant’s current state of residence. This potential misclassification could attenuate observed associations with state governmental leaning. However, despite this limitation, we still observed an independent association between state governorship and discrimination, thus suggesting that even a relatively short residence in a given state may be sufficient to experience such discrimination.

Lastly, state governmental leaning was also operationalized as a binary measure based on the political affiliation of the sitting governor during the study period. This could oversimplify the complexity of state political environments, for example in swing states where party leadership changes over time. The political climates and structural conditions that influence healthcare access are shaped by long-term policy histories and sociocultural factors that may not shift abruptly with changes in gubernatorial affiliation. As such, our measure may not fully capture the total political context experienced by participants. In contrast, the perceptions of homophobia within the sexual minority population may be exacerbated given a populous vote for a political party not largely in favor of LGBTQ + rights and heighten the microaggressions in the healthcare delivery system. As such, perceptions of discrimination and heightened psychosocial responses may result from an elected Governor’s political affiliation alone and not necessarily through long-term structural practices.

## Public health implications

These findings underscore the importance of fostering equitable, inclusive survivorship care for sexual minority populations. Healthcare systems should prioritize efforts to build affirming clinical environments that reduce stigma and discrimination, which can deter SM survivors from seeking care or disclosing their identities. Public health initiatives that strengthen social cohesion and address psychosocial stressors may improve long-term well-being and treatment adherence among SM cancer survivors.

At the policy level, addressing structural and contextual factors such as political and social environments that perpetuate disparities is essential. Partnerships between clinicians, researchers, and policymakers can help translate evidence into interventions that ensure all survivors, regardless of sexual orientation or geographic context, receive supportive and nondiscriminatory care. Continued research using large, diverse datasets such as the *All of Us* Research Program can guide these equity-driven strategies and inform national efforts to reduce psychosocial and structural barriers to survivorship care.

## Conclusion

The AoU Research Program data allowed us to investigate the independent associations between sexual minority status and different types of stressors among a large sample of cancer survivors. We further explored whether these associations differed by state governmental leaning. We found that identifying as a sexual minority is associated with increased likelihood of experiencing any DMS, medium/high PS, and low NSC, compared to their heterosexual counterparts. Additionally, differences by state governmental leaning were observed in the association between sexual minority status and DMS. Sexual minority cancer survivors residing in Republican-leaning states had higher odds of experiencing any DMS compared with heterosexual survivors, whereas no significant differences were observed among those living in Democratic-leaning states. These findings may help guide healthcare systems, particularly in states where disparities may be more pronounced, to promote inclusivity and acceptance of sexual minority survivors, thereby minimizing the potential to forgo medical care due to prior discriminatory experiences.

## Supplementary Information

Below is the link to the electronic supplementary material.

Supplementary file1 (DOCX 31 KB)

Supplementary file2 (DOCX 21 KB)

## Data Availability

The dataset supporting the conclusions of this article is available in the NIH All of Us Research Program repository (https://www.researchallofus.org/). Access to the data requires approval and registration through the All of Us Researcher Workbench (https://workbench.researchallofus.org/). For code, please email the corresponding author.
