# Supplementary material for: Disparities in multidimensional psychosocial stressors by sexual identity among cancer survivors from the All of Us Research Program
Source: Cancer Causes Control. 2026 Apr 3;37(5):71. doi: 10.1007/s10552-026-02157-w (PMC13048929; doi:10.1007/s10552-026-02157-w)
Supplement: Supplementary file 1 — Supplementary file1 (DOCX 31 KB) [file 10552_2026_2157_MOESM1_ESM.docx]

| Supplementary Table 1: Unadjusted Associations Between Disaggregated Sexual Minority Identity and Psychosocial Stressors Among Cancer Survivors, All of Us Research Program, United States, 2018–2022 | | | |
| --- | --- | --- | --- |
|  | Any Discrimination in Medical Settings | Medium/High Perceived Stress | Low Neighborhood Social Cohesion |
|  | OR (95% CI) | OR (95% CI) | OR (95% CI) |
| Heterosexual | Ref | Ref | Ref |
| Bisexual | **2.21 (1.66-3.00)** | **2.67 (2.13-3.38)** | **2.21(1.77-2.79)** |
| Gay | 1.07 (0.86-1.35) | **1.51 (1.23-1.85)** | **2.13 (1.72-2.64)** |
| Lesbian | **2.08 (1.41-3.19)** | **1.59 (1.18-2.16)** | 1.13 (0.84-1.52) |
| Other | 2.65 (0.91-11.24) | 2.14 (0.93-5.31) | **3.83 (1.53-11.61)** |
| OR = Odds Ratios, CI = confidence interval, Ref = Reference  Bolded represents statistical significance  “Other” includes individuals who self-identified as queer, asexual, two-spirit, polysexual, omnisexual, sapiosexual, or pansexual. This grouping follows All of Us privacy requirements to protect participant identity (no cells with n < 20 displayed). | | | |
