# Supplementary material for: Disparities in multidimensional psychosocial stressors by sexual identity among cancer survivors from the All of Us Research Program
Source: Cancer Causes Control. 2026 Apr 3;37(5):71. doi: 10.1007/s10552-026-02157-w (PMC13048929; doi:10.1007/s10552-026-02157-w)
Supplement: Supplementary file 2 — Supplementary file2 (DOCX 21 KB) [file 10552_2026_2157_MOESM2_ESM.docx]

| Supplemental Table 2: Multivariable Associations Between Disaggregated Sexual Minority Identity and Psychosocial Stressors Among Cancer Survivors, All of Us Research Program, United States, 2018–2022 | | | |
| --- | --- | --- | --- |
|  | Any Discrimination in Medical Settings^a^ | Medium/High Perceived Stress^b^ | Low Neighborhood Social Cohesion^c^ |
|  | aOR (95% CI) | aOR (95% CI) | aOR (95% CI) |
| Heterosexual | Ref | Ref | Ref |
| Bisexual | **1.67 (1.25-2.29)** | **2.22 (1.75-2.82)** | **1.86 (1.48-2.35)** |
| Gay | 0.90 (0.71-1.14) | **1.38 (1.12-1.70)** | **2.04 (1.65-2.54)** |
| Lesbian | **1.91 (1.28-2.95)** | **1.42 (1.04-1.95)** | 1.00 (0.74-1.36) |
| Other | 2.00 (0.67-8.64) | 1.59 (0.68-4.02) | **3.34 (1.32-10.22)** |
| Notes:  Adjusted models  ^a^neighborhood social cohesion and perceived stress, ^b^discrimination in medical settings and neighborhood social cohesion, ^c^discrimination in medical settings and perceived stress  aOR = adjusted Odds Ratios, CI = confidence interval , Ref = Reference  Bolded represents statistical significance  “Other” includes individuals who self-identified as queer, asexual, two-spirit, polysexual, omnisexual, sapiosexual, or pansexual. This grouping follows All of Us privacy requirements to protect participant identity (no cells with n < 20 displayed). | | | |
